# Buspirone Enhances Cell Survival and Preserves Structural Integrity during Oxidative Injury to the Retinal Pigment Epithelium

**DOI:** 10.3390/antiox12122129

**Published:** 2023-12-17

**Authors:** Manas R. Biswal, Ryan J. Paulson, Riddhi Vichare, Alfred S. Lewin

**Affiliations:** 1 Department of Pharmaceutical Sciences, USF Taneja College of Pharmacy, Tampa, FL 33612, USAvicharer@usf.edu (R.V.); 2 Department of Molecular Genetics & Microbiology, College of Medicine, University of Florida, Gainesville, FL 32603, USA; lewin@ufl.edu

**Keywords:** retina, buspirone, oxidative stress, retinal pigment epithelium, antioxidants, ZO-1, ARPE-19, NaIO_3_

## Abstract

Chronic oxidative stress impairs the normal functioning of the retinal pigment epithelium (RPE), leading to atrophy of this cell layer in cases of advance age-related macular degeneration (AMD). The purpose of our study was to determine if buspirone, a partial serotonin 1A (5-HT1A) receptor agonist, protected against oxidative stress-induced changes in the RPE. We exposed differentiated human ARPE-19 cells to paraquat to induce oxidative damage in culture, and utilized a mouse model with sodium iodate (NaIO_3_)-induced oxidative injury to evaluate the effect of buspirone. To investigate buspirone’s effect on protective gene expression, we performed RT–PCR. Cellular toxicities and junctional abnormalities due to paraquat induction in ARPE-19 cells and buspirone’s impact were assessed via WST-1 assays and ZO-1 immunostaining. We used spectral-domain optical coherence tomography (SD-OCT) and ZO-1 immunostaining of RPE/choroid for structural analysis. WST-1 assays showed dose-dependent protection of viability in buspirone-treated ARPE-19 cells in culture and preservation of RPE junctional integrity under oxidative stress conditions. In the NaIO_3_ model, daily intraperitoneal injection (i.p.) of buspirone (30 mg/kg) for 12 days improved the survival of photoreceptors compared to those of vehicle-treated eyes. ZO-1-stained RPE flat-mounts revealed the structural preservation of RPE from oxidative damage in buspirone-treated mice, as well as in buspirone-induced *Nqo1*, *Cat*, *Sqstm1*, *Gstm1*, and *Sod2* genes in the RPE/choroid compared to untreated eyes. Since oxidative stress is implicated in the pathogenesis AMD, repurposing buspirone, which is currently approved for the treatment of anxiety, might be useful in treating or preventing dry AMD.

## 1. Introduction

Age-related macular degeneration (AMD) is a prevalent and debilitating ocular disease that affects millions of individuals worldwide, leading to progressive central vision loss and a substantial burden on healthcare systems. The hallmark of AMD is the degeneration of the retinal pigment epithelium (RPE), a critical cell monolayer essential for maintaining retinal health. While the pathogenesis of AMD is multifactorial, oxidative stress and chronic inflammation are pivotal contributors [1,2], making them compelling targets for therapeutic intervention.

Buspirone, a widely recognized anxiolytic agent [3,4,5], has recently garnered significant attention in the realm of ophthalmology due to its potential to address a critical unmet need in the treatment of age-related macular degeneration (AMD) [6]. This innovation lies in buspirone’s distinctive pharmacological profile, which includes serotonin 1A (5-HT1A) receptor agonism alongside antioxidant and neuroprotective properties. This combination positions buspirone as a multifaceted candidate for retinal therapy. Studies have shown that 5-HT1A receptor activation can exert neuroprotective effects in various neuronal systems [7,8,9], and emerging evidence suggests it has relevance to the retina, particularly in contexts involving oxidative stress and neuroinflammation. Moreover, buspirone has exhibited robust antioxidant capabilities in preclinical models [10,11] aiding in the attenuation of oxidative damage, a pivotal hallmark of AMD pathogenesis [12,13].

The retinal pigment epithelium (RPE) serves as a vital cellular barrier and orchestrates critical functions essential for the maintenance of retinal homeostasis and visual function. In the face of oxidative stress and the constant challenge posed by environmental factors, the RPE relies on a sophisticated network of protective genes to safeguard its integrity and ensure optimal functionality. Among these, the genes NAD(P)H dehydrogenase quinone 1 (*Nqo1*), catalase (*Cat*), Sequestosome 1 (*Sqstm1*), glutathione S-transferase M1 (*Gstm1*), and superoxide dismutase 2 (*Sod2*) have emerged as pivotal guardians, actively participating in the defense against various insults. This introduction seeks to elucidate why the expression and functionality of these genes are crucial to the protective milieu within the RPE.

*Nqo1*, a multifunctional enzyme and a targeted gene of transcription factor nuclear factor erythroid-2-related factor 2 Nfe2l2 (also known as Nrf2), plays a central role in the detoxification of reactive quinones and the maintenance of cellular redox balance [14]. Its ability to neutralize electrophilic compounds and mitigate oxidative stress positions *Nqo1* as a crucial guardian against cellular damage in the RPE [15]. By preventing the accumulation of harmful species, *Nqo1* acts as an indispensable component of the RPE’s defense mechanism [16]. Catalase, an enzymatic cornerstone in the breakdown of hydrogen peroxide, assumes a paramount role in mitigating oxidative damage within the RPE. As a primary defender against reactive oxygen species (ROS), catalase serves to preserve the delicate equilibrium of the cellular microenvironment, preventing oxidative stress-induced harm and bolstering the resilience of the RPE. Sqstm1, a regulator of autophagy and cellular quality control, emerges as a key player in maintaining RPE homeostasis. Its involvement in the clearance of damaged cellular components and misfolded proteins positions *Sqstm1* as a safeguard against the deleterious consequences of cellular stress. Via the orchestration of autophagic processes, Sqstm1 contributes to the overall protection and longevity of RPE cells.

*Gstm1*, an integral member of the glutathione S-transferase family, plays a pivotal role in detoxification processes within the RPE. By catalyzing the conjugation of electrophilic compounds with glutathione, *Gstm1* enhances cellular defense against oxidative stress and maintains the integrity of critical cellular components [17]. Its presence in the RPE improves efficient detoxification mechanisms with oxidative damage and enhances RPE protection. *Sod2*, a mitochondrial antioxidant enzyme, serves as a frontline defender against superoxide radicals within the RPE [18]. By catalyzing the dismutation of superoxide into oxygen and hydrogen peroxide, *Sod2* acts as a critical component of the RPE’s antioxidant arsenal. Its capacity to neutralize one of the primary reactive oxygen species reinforces the importance of *Sod2* in preserving redox balance and cellular viability in the RPE [19]. These protective genes weave a complex yet interdependent web of defense mechanisms crucial for the RPE’s ability to withstand oxidative stress and maintain its pivotal role in retinal health.

Previous research has demonstrated the efficacy of 5-HTA1 receptor agonists in mitigating oxidative stress in diverse cellular and animal models [20,21,22,23], offering a solid foundation for the application of buspirone in retinal protection. Additionally, its favorable safety profile and established clinical use in psychiatry underscore its potential to be repurposed in ophthalmology, offering an expedited pathway for clinical translation. The convergence of these factors presents an opportunity to explore buspirone’s therapeutic potential in preserving retinal function and combating AMD, with implications for patients worldwide.

Paraquat and sodium iodate (NaIO_3_) are both chemical agents used to induce oxidative stress in cell culture and animal models, respectively, for studying retinal degeneration [22,24]. However, it is important to note that they are distinct agents with different mechanisms of action and applications. While paraquat-induced cell models and NaIO_3_-induced mouse models are distinct, they can be used in complementary ways. Paraquat-induced cell models can provide insights into the cellular and molecular responses of retinal cells to oxidative stress, which is a component of the broader retinal degeneration process. Understanding how oxidative stress affects cellular responses in vitro (paraquat model) in response to a therapeutic agent can inform the interpretation of the in vivo effects observed in NaIO_3_-induced mouse models. The paraquat model may be useful for studying early responses to oxidative stress at the cellular level, while the NaIO_3_-induced mouse model allows for the investigation of downstream consequences in the context of the intact retina and interactions between different retinal cell types [25]. The relationship lies in their shared focus on oxidative stress and their potential synergy in providing a more comprehensive view of retinal pathology and potential interventions.

In this study, we tested an well-known anxiolytic drug in mitigating RPE atrophy using a two-tiered approach. First, we investigated the effects of buspirone in ARPE-19 cells, an established in vitro model for RPE function, to understand its impact on cell viability and protective mechanisms against oxidative stress. ARPE-19 cells, derived from the human RPE, offer valuable insights into the cellular aspects of AMD pathology, and serve as a practical platform for drug testing [26,27]. Subsequently, we translated our findings to an in vivo setting by employing a mouse model of RPE atrophy induced using sodium iodate (NaIO_3_). The NaIO_3_ model is characterized by oxidative stress-mediated damage to the RPE, closely mimicking certain features of AMD [28,29,30,31]. Buspirone was administered to assess its potential in ameliorating retinal degeneration, evaluating its suitability as a therapeutic agent.

By adopting this dual approach, we aim to provide a comprehensive evaluation of buspirone’s efficacy in preserving RPE function and retinal health. Our findings hold promise for the development of novel therapies to combat AMD, potentially offering renewed hope for individuals afflicted by this devastating ocular disease.

## 2. Materials and Methods

### 2.1. Cell Culture

ARPE-19 cells (Cat no: CRL-2302™), derived from the human retinal pigment epithelium, were purchased from ATCC (Manassas, VA, USA) and maintained in our laboratory according to established protocols [32,33]. Cells were cultured in T75 flasks using Dulbecco’s Modified Eagle Medium (DMEM)/F-12 supplemented with 10% fetal bovine serum (FBS) and 1% penicillin–streptomycin solution (ATCC, Manassas, VA, USA). The culture medium was changed every 2–3 days, and cells were maintained in a humidified incubator at 37 °C with 5% CO_2_. Subculturing was performed when cells reached 80–90% confluency, using a trypsin-EDTA solution to detach the cells from the flask. For experimental assays, ARPE-19 cells were seeded at an appropriate density in 6-well plates or culture dishes, depending on the specific requirements of the assay. All experiments were conducted using cells between passages 4 and 10 to ensure consistency and minimize potential phenotypic drift. To obtain RPE monolayers with cobblestone appearance and tight junctions, ARPE-19 cells sere cultured in Minimum Essential Medium including nicotinamide, as described by Hazim et al. [26].

### 2.2. Cellular Apoptosis Assay

Cell viability was assessed using the cell proliferation reagent (WST-1, Cat no: 05015944001, Germany) [34,35] to evaluate the impact of buspirone on ARPE-19 cells. The WST-1 assay protocol is based on the cleavage of the tetrazolium salt WST-1 to formazan using cellular mitochondrial dehydrogenases. Briefly, ARPE-19 cells were seeded in 96-well plates at a density of 6000 cells per well and allowed to adhere overnight. Following adherence, cells were treated with varying concentrations of buspirone and paraquat for a specified duration. Control wells received equivalent volumes of the vehicle solution. After treatment, 10 μL of WST-1 reagent was added to each well, and the plates were incubated for an additional two hours at 37 °C. Absorbance was measured at 450 nm using a microplate reader. Data were analyzed using GraphPad-Prism (version 9), and viability percentages were calculated relative to vehicle-treated controls. All experiments were conducted in triplicate and repeated at least three times to ensure statistical significance.

### 2.3. Animals

Male C57BL/6J mice, aged 6–8 weeks, were utilized in accordance with University of South Florida (USF) Institutional Animal Care and Use Committees (IACUCs) for animal care and use. To induce retinal degeneration, a sodium iodate (NaIO_3_) model was employed. Mice were randomly assigned to experimental groups: the NaIO_3_-treated group and the NaIO_3_ + buspirone-treated group. NaIO_3_ was administered via a single intraperitoneal injection at a dosage of 20 mg/kg body weight. Buspirone treatment commenced three days before NaIO_3_ injection, with daily intraperitoneal administrations at a dosage of 30 mg/kg body weight for next 12 days post NaIO_3_ injection. Control animals received equivalent volumes of the vehicle solution. SD-OCT and RPE/Choroid flat mount assessments were conducted one day after the last buspirone treatment to evaluate the efficacy of buspirone in ameliorating retinal degeneration induced by NaIO_3_.

### 2.4. Real Time PCR

Our investigation was designed to assess gene expression changes within the retinal pigment epithelium (RPE) and choroidal tissues. To investigate the potential therapeutic effects of buspirone, RPE and choroid tissues were carefully dissected, and total RNA was extracted using the RNeasy Mini Kit (Qiagen, MD, USA). The resulting RNA samples were then reverse-transcribed into complementary DNA (cDNA), quantitative real-time PCR was performed using CFX Opus 96 Real-Time PCR System (Bio-Rad, CA, USA) [36], and its program was used to analyze the expression levels of key genes implicated in retinal degeneration. The PCR primers used for this analysis are listed in Table 1.

### 2.5. SD-OCT Imaging and Measurement of Total Retinal Thickness

Spectral-domain optical coherence tomography (SD-OCT) imaging with the Phoenix Micron IV system was used to assess retinal morphology in our study [37]. Both male and female C57BL/6 mice, aged 8–10 weeks, were selected for this investigation. After appropriate anesthesia and pupil dilation, mice were gently positioned, and SD-OCT scans were obtained with high resolution and precision. Specifically, we focused on measuring total retinal thickness, which encompasses the entire retinal structure, from the inner limiting membrane to the retinal pigment epithelium. Multiple scans were acquired per eye to ensure data accuracy and consistency. The acquired images were subsequently processed and analyzed using the intelligent eye segmentation software “InSight” (version -2) to visualize retinal layers automatically or interactively to obtain precise measurements of total retinal thickness. This approach provided a detailed assessment of retinal structural alterations, offering valuable insights into the effects of drugs or oxidants on retinal integrity.

### 2.6. Immunohistochemistry

For the assessment of tight junction integrity, differentiated ARPE-19 cells [26] were grown on coverslips and fixed with 4% paraformaldehyde [38]. After permeabilization with 0.3% Triton X-100, cells were incubated with a primary antibody against ZO-1 (Invitrogen, Waltham, MA, USA) (Cat. No-40-2200, 1:200 ratio) overnight at 4 °C, followed by Alexa Fluor 488 goat anti-rabbit (Molecular Probes; Invitrogen, Waltham, MA, USA) (Cat no: A11008) secondary antibody incubation (1:1000) for an 1 h. Imaging was performed using a Keyence fluorescence microscope imaging system. For the examination of ZO-1 expression in retinal pigment epithelium (RPE) and choroidal tissues, enucleated mouse eyes were dissected to obtain RPE/choroid flat mounts. These were fixed, blocked, and incubated with the same primary antibody against ZO-1 (1:200) overnight at 4 °C [39]. Subsequently, tissues were treated with DyLight 650 conjugated goat anti-rabbit secondary antibody (Invitrogen, Waltham, MA, USA) (Cat no: SA5-10041, 1:1000). Flat mounts were mounted on glass slides and visualized using a Keyence fluorescence microscope imaging system. This dual approach allowed for a comprehensive evaluation of ZO-1 expression patterns both at the cellular level in ARPE-19 cells and within intact RPE/choroid tissues from mice [40].

### 2.7. Statistical Analysis

For experiments involving ARPE-19 cell cultures, results were expressed as mean ± standard deviation (SD) of three independent experiments. Statistical significance was determined using Student’s *t*-test (two-tailed) using GraphPad Prism software (version 9). For assessments involving C57BL/6J mice and RPE/choroid flat mounts, data were analyzed using GraphPad Prism. Differences between the two groups were evaluated using Student’s *t*-test (two-tailed). Results were considered statistically significant when *p* < 0.05. All statistical analyses were performed by an investigator blinded to the experimental conditions to minimize bias.

## 3. Results

### 3.1. Buspirone Prevented Oxidative Stress Induced Cell Death

**Figure 1:**
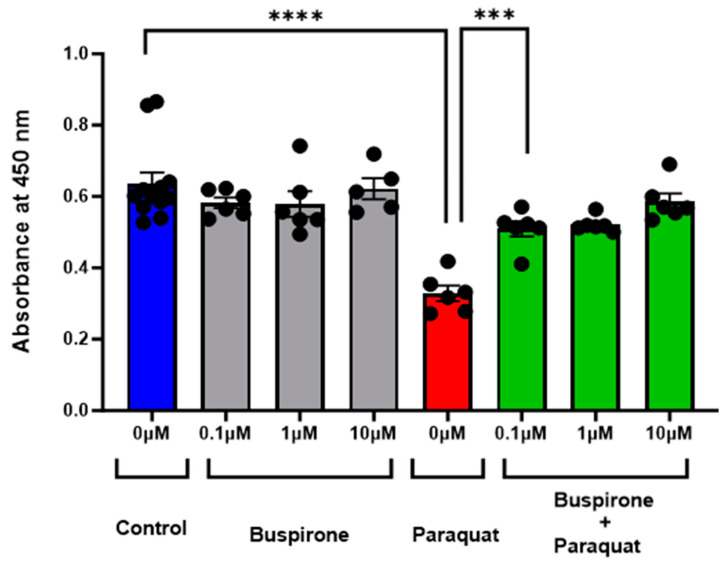
**Effect of Buspirone Pretreatment on Paraquat-Induced Cell Death in ARPE-19 Cells as Assessed via WST-1 Assay.** The graph depicts the absorbance values obtained via WST-1 assay, reflecting cell viability, in ARPE-19 cells subjected to various treatments. The x-axis represents different treatment groups: (1) control, (2) buspirone-treated, (3) induced cell death, and (4) buspirone pre-treatment + paraquat. The y-axis denotes the absorbance measured at 450 nm. Data points represent the mean ± standard error of mean of n = 6, with 3 independent experiments. Statistical significance was determined using Student’s *t*-test between two groups, with *p* < 0.05 considered as statistically significant. *** *p* < 0.001, **** *p* < 0.0001.

In our investigation, the potential of buspirone in mitigating oxidative stress-induced cell death was evaluated utilizing ARPE-19 cells, an established in vitro model for retinal pigment epithelium (RPE) function. Following exposure to paraquat (at 400 µM), which induces mitochondrial oxidative stress, cells treated with buspirone exhibited a pronounced increase in viability. Treatment with buspirone, at concentrations ranging from 0.1 µM to 10 µM, conferred a significant protective effect against oxidative stress-induced cell death compared to untreated controls (Figure 1). The results, assessed via WST-1 assay, revealed a robust preservation of cell viability, indicative of buspirone’s potent cytoprotective properties in the face of oxidative insult.

### 3.2. Buspirone Prevented RPE Structural Disintegration from Oxidative Damage

**Figure 2:**
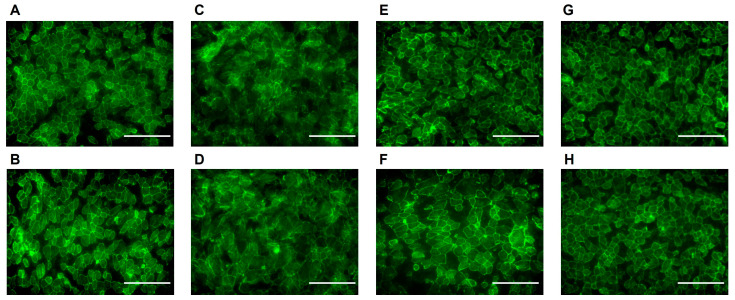
**Immunocytochemical Staining of Differentiated ARPE-19 Cells Treated with Paraquat and Buspirone.** The images display the immunocytochemistry of differentiated ARPE-19 cells subjected to different treatments. ZO-1, a marker for tight junctions, is labeled in green. (**A**,**B**) Control group, (**C**,**D**) paraquat (1000 μM)-induced cell stress, (**E**,**F**) buspirone (1 μM)-pre-treated cells followed by paraquat (1000 μM) exposure, and (**G**,**H**) buspirone (10 μM) pre-treated cells followed by paraquat (1000 μM) exposure. (40× magnification, scale bar 100 micron).

To assess the protective effects of buspirone on differentiated ARPE-19 cells, we employed ZO-1 immunocytochemistry on cells cultured in 8-well chamber slides. Following differentiation [26], ARPE-19 cells were subjected to oxidative stress induced using paraquat (1000 µM) treatment. Strikingly, cells pre-treated with buspirone demonstrated a substantial preservation of structural integrity compared to untreated counterparts (Figure 2). ZO-1, a critical tight junction protein, exhibited a robust and organized distribution pattern along the cell–cell boundaries, indicative of maintained cellular architecture. Conversely, in the absence of buspirone, paraquat-induced oxidative stress led to a noticeable disruption of ZO-1 staining, reflecting compromised cell–cell interactions and structural disintegration. These findings underscore the potent protective effect of buspirone in shielding differentiated ARPE-19 cells against oxidative damage, highlighting its potential as a therapeutic candidate for retinal degenerative conditions characterized by heightened oxidative stress.

### 3.3. Presence of Buspirone-Preserved Retinal Structure in an Acute Model of RPE Oxidative Damge

**Figure 3:**
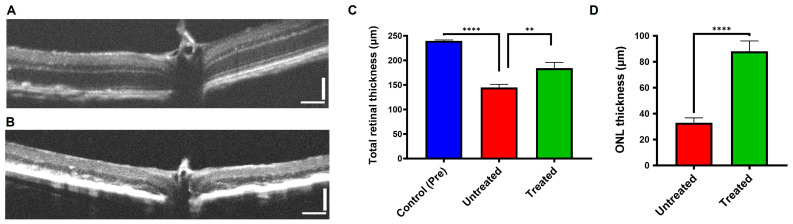
**Retinal Thickness Analysis in Response to Buspirone Treatment**. The figure illustrates the impact of buspirone treatment on retinal structure in a NaIO_3_-induced RPE injury model at the 12-day time point. Representative SD-OCT images depict the structural changes in the retinal layers in response to buspirone treatment (**A**). An image from a NaIO_3_ injected mouse without buspirone treatment (**B**) is shown for comparison. (**C**) Total retinal thickness was measured, indicating the effects of buspirone treatment on overall retinal architecture. (**D**) Measurement of the outer nuclear layer quantifies the impact of buspirone on photoreceptor survival. Data represent the mean ± s.e.m. (Control pre-treatment, n= 20; untreated, n = 9; treated, n = 11). ** *p* < 0.01, **** *p* < 0.0001. (Scale bar 75 micron).

Utilizing spectral-domain optical coherence tomography (SD-OCT), we assessed the impact of buspirone on the preservation of retinal structure in an acute model of retinal pigment epithelium (RPE) oxidative damage (Figure 3 and Figure S1). The measurements of total retinal thickness and outer nuclear layer (ONL) thickness provided insights into the integrity of the retinal architecture. Strikingly, the presence of buspirone conferred a preservation of both total retinal thickness and ONL thickness. Notably, the photoreceptor layer, a critical component of retinal function, exhibited a significant conservation, emphasizing buspirone’s protective effect. These findings underscore the potency of buspirone in mitigating acute RPE oxidative damage, leading to the preservation of retinal layers and, particularly, the photoreceptor layer, which holds significance for vision maintenance.

### 3.4. Buspirone Preserved RPE Structural Integrity In Vivo from Oxidative Damage

**Figure 4:**
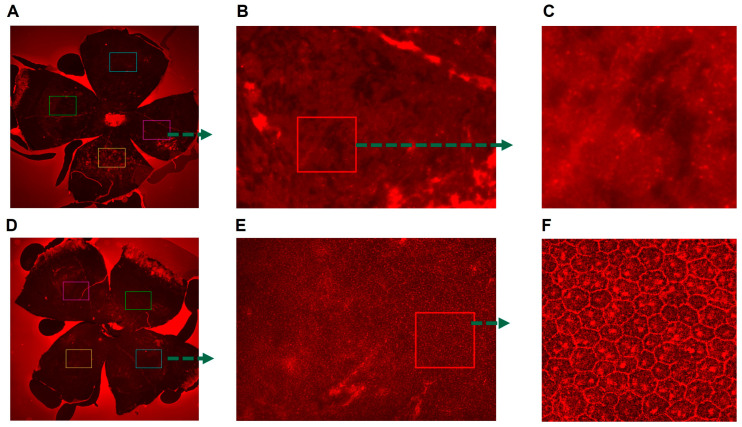
**Buspirone Preserves RPE Structural Integrity In Vivo: RPE Flat Mount Immunostaining for ZO-1**. The images demonstrate the effects of buspirone treatment on RPE (Retinal Pigment Epithelium) structural integrity in vivo. (**A**) Mice injected with NaOI3 but untreated with buspirone served as a comparison (4× magnification). (**B**) 20× magnification, (**C**) 100× magnification, (**D**) The buspirone treated mouse showed preserved RPE morphology in response to buspirone administration (4× magnification). (**E**) 20× magnification. (**F**) 100× magnificationThe RPE choroid tissues were processed for immunostaining for ZO-1 (Zonula Occludens-1) and red fluorescence is visualized using fluorescence microscopy, highlighting tight junctions within the RPE.

The potential of buspirone in preserving retinal structure was evaluated in an acute model of retinal pigment epithelium (RPE) oxidative damage, using RPE/choroidal flat mount assessments coupled with ZO-1 staining. Following exposure to NaIO_3_, a striking contrast in retinal structural integrity became evident. In the presence of buspirone, the retinal flat mounts displayed a well-preserved and -organized arrangement of hexagonal cells, characteristic of healthy RPE tissue, as observed via fluorescent imaging (Figure 4). The ZO-1 staining patterns further emphasized the intact cellular architecture, with the critical tight junction protein maintaining its well-defined distribution. Conversely, in the absence of buspirone, oxidative damage induced using the acute model led to a distinct disarray of hexagonal cells, indicative of structural damage. ZO-1 staining corroborated these findings by highlighting disrupted cell–cell interactions. These results underscore the robust retinal-preserving potential of buspirone, offering promising prospects for its application in retinal degenerative conditions characterized by oxidative stress.

### 3.5. Increased Expression of Antioxidant Genes in the RPE/Choroid in Response to Buspirone Treatment

**Figure 5:**
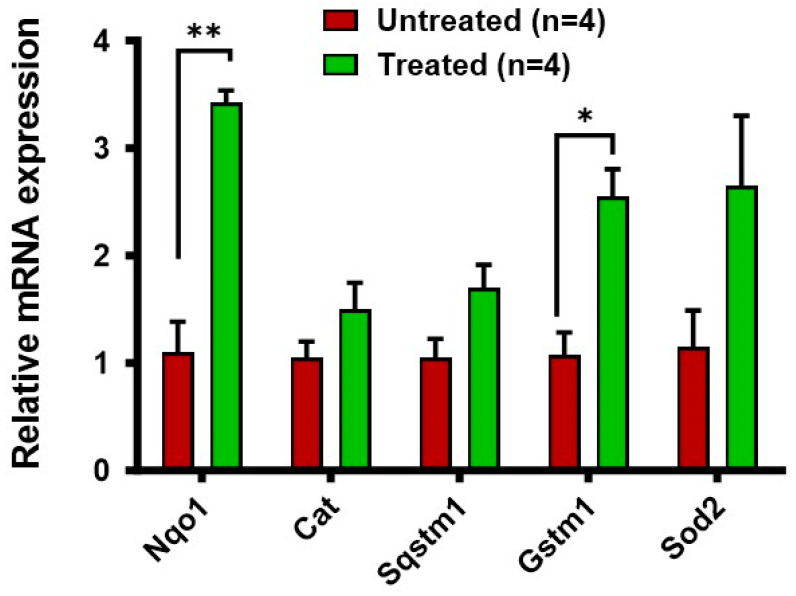
**Relative mRNA Expression of Antioxidant Genes in RPE/Choroid: Buspirone-Treated Eyes vs Untreated Control Eyes.** The graph illustrates the comparative mRNA expression levels of key antioxidant genes in the RPE/choroid complex. Buspirone-treated eyes are represented in green, while untreated control eyes are indicated in red. Data are normalized to *beta-actin* and presented as fold change relative to control. Antioxidant genes analyzed include *Nqo1*, *Cat*, *Sqstm1*, *Gstm1*, and *Sod2*, known for their pivotal role in oxidative stress response. Data represents the mean ± standard error of the mean (SEM) of n = 4. Statistical significance was determined using Student’s *t*-test between two groups, with *p* < 0.05 considered as statistically significant. * *p* < 0.05, ** *p* < 0.01.

To explore the impact of buspirone treatment on the expression of key antioxidant genes within the retinal pigment epithelium (RPE) and choroidal tissues, we used reverse transcription–polymerase chain reaction (RT-PCR) to analyze the expression profiles of the antioxidant enzymes *Nqo1*, *Sod2*, *Gstm1*, and *Cat* [21] (Figure 5). Remarkably, buspirone treatment led to a significant and robust upregulation of these antioxidant genes compared to control groups. *Nqo1*, a critical player in cellular defense against oxidative stress, exhibited a substantial increase in expression. Similarly, the expression of *Sod2* and *Gstm1*, crucial components of the antioxidant defense network, displayed marked elevations. In a separate experiment, we also observed a dose-dependent and elevated trend of expression of key antioxidant genes in ARPE-19 cells under normal conditions (Figure S2).

## 4. Discussion

### 4.1. Buspirone’s Multifaceted Protective Effects in Retinal Health

The results of our study reveal a multifaceted potential for buspirone in preserving retinal health under conditions of oxidative stress. Our findings in differentiated ARPE-19 cells demonstrate a substantial protective effect against oxidative damage, evident in the preservation of structural integrity as assessed via ZO-1 immunocytochemistry. This effect extended to an acute model of retinal pigment epithelium (RPE) oxidative damage, where buspirone treatment led to a pronounced conservation of total retinal thickness and outer nuclear layer (ONL) thickness, crucial indicators of retinal structural integrity. Notably, the heightened preservation of the photoreceptor layer highlights the therapeutic promise of buspirone in conditions characterized by retinal degeneration. The observed increase in expression of antioxidant genes, including *Nqo1*, *Sod2*, and Gstm1, within the RPE/choroid further underscores buspirone’s capacity to bolster cellular defense mechanisms against oxidative stress. Collectively, these findings paint a compelling picture of buspirone as a potent candidate for therapeutic intervention in retinal degenerative conditions, offering a multi-pronged approach to combat oxidative stress-induced damage.

### 4.2. Potential Mechanisms Underlying Buspirone’s Protective Effects

The mechanisms underlying buspirone’s protective effects in the retina merit further exploration. Buspirone’s 5-HT1A receptor agonism has been implicated in neuroprotection in various neuronal systems, suggesting a potential neuroprotective role in the retina. Additionally, the observed increase in expression of antioxidant genes may contribute to the heightened cellular defense against oxidative stress. *Nqo1* is a key player in the cellular response to oxidative damage [41], and its upregulation may signify a critical aspect of buspirone’s mechanism of action. These results underscore the potency of buspirone in inducing the upregulation of antioxidant genes in the RPE/choroid, indicative of its capacity to bolster the cellular defense mechanisms against oxidative stress. It is important to note that the exact mechanisms by which buspirone influences antioxidative gene expression in RPE cells may involve multiple pathways and factors. The molecular details of buspirone’s actions in the context of retinal protection are an active area of research and may involve intricate interactions within the cellular environment. Further studies, including molecular analyses and gene expression profiling, are needed to elucidate the specific pathways and genes influenced by buspirone in the RPE and to establish a more comprehensive understanding of its antioxidative effects. Furthermore, the potential interplay between buspirone’s 5-HT1A receptor agonism and its antioxidant properties warrants investigation in other models of retinal or RPE atrophy, as it may shed light on the synergistic mechanisms underlying its protective effects.

### 4.3. Clinical Implications and Future Directions

The promising outcomes of our study hold significant clinical implications for the treatment of age-related macular degeneration (AMD) and other retinal degenerative conditions characterized by oxidative stress [42,43]. Buspirone’s primary pharmacological use has been as an anxiolytic agent, and its potential therapeutic effects in the field of ophthalmology are an emerging area of research. Buspirone’s demonstrated capacity to preserve retinal structure and enhance antioxidant defense mechanisms positions it as a potential game-changer in the field of retinal therapeutics. Furthermore, its well-established safety profile in psychiatric applications provides a solid foundation for expedited clinical translation. Future studies may delve deeper into the specific molecular pathways activated via buspirone in the retina and explore potential synergistic effects with other therapeutic agents. Urbina and colleagues have reported that a 5HT1a agonist (5OH-DPAT) produces a dose dependent increase in cAMP in the goldfish retina, and they suggest that the impact of 5HT1a agonists is mediated via cAMP-regulated pathways [44]. Additionally, investigations into the long-term effects of buspirone treatment on retinal health and function will be crucial for a comprehensive understanding of its therapeutic potential in retinal degenerative diseases. There has been no widespread exploration of buspirone specifically in recent studies or clinical trials evaluating buspirone in the context of other eye diseases, including glaucoma. Given buspirone’s potential antioxidative and neuroprotective effects, it could be of interest to study conditions involving retinal degeneration or optic nerve damage, and our future studies will investigate it.

### 4.4. Limitations of the Study

While our study provides compelling evidence for buspirone’s therapeutic potential, certain limitations must be acknowledged and will be explored in our future studies. Firstly, our experiments were primarily conducted in cell culture and in acute in vivo models, and as such, the long-term effects of buspirone treatment in chronic conditions require further investigation. Our study did not provide enough information related to retention of buspirone following i.p. delivery of buspirone and RNA changes of *Nqo1*, *Cat*, *Sqstm1*, *Gstm1*, and *Sod2* genes in RPE without NaIO_3_ treatment. We are aiming for future studies where we will investigate RNA and proteome changes in response to buspirone treatment in retinal cells in presence or absence of oxidants. Additionally, the specific dose–response relationships and optimal treatment durations necessitate refinement in future studies. Moreover, while our focus was primarily on the antioxidant properties of buspirone, other potential mechanisms of action may contribute to its observed effects, warranting comprehensive exploration. Lastly, clinical translation will require rigorous evaluation of safety and efficacy in human subjects, mandating further preclinical and clinical investigations.

## 5. Conclusions

In conclusion, our study illuminates a groundbreaking avenue in the pursuit of effective treatments for retinal degenerative disorders. Via a comprehensive assessment of buspirone’s multifaceted effects, we have uncovered its remarkable potential in preserving retinal structure and fortifying cellular defenses against oxidative stress. This innovative approach, harnessing the synergistic power of buspirone’s 5-HT1A receptor agonism and potent antioxidant properties, represents a promising breakthrough in retinal therapeutics. By targeting not only the structural integrity of the retina but also its underlying cellular resilience, buspirone holds the promise of transformative interventions for conditions like age-related macular degeneration. The safety and established clinical usage of buspirone further expedites its potential translation to clinical settings, opening a new chapter in the treatment of retinal degenerative disorders. As we stand at the precipice of a new era in ophthalmology, the potential of buspirone offers renewed hope for those affected by these devastating conditions, paving the way for a brighter future in retinal health.

## Figures and Tables

**Table 1:** Essential details for the genes analyzed, including their respective gene names, RefSeq IDs, forward primer sequences, and reverse primer sequences. These primer sequences are crucial for the accurate amplification of the target genes in real time PCR experiments.

Genes	Ref. Seq	Forward Primer	Reverse Primer
*Beta-Actin*	NM_007393.3	CGAGCACAGCTTCTTTGCAG	TTCCCACCATCACACCCTGG
*Nqo1*	NM_008706.5	CGACAACGGTCCTTTCCAGA	CCAGACGGTTTCCAGACGTT
*Cat*	NM_009804.2	CGCAATCCTACACCATGTCG	AGTATCCAAAAGCACCTGCTCC
*Sqstm1*	NM_011018.2	GGAAGCTGCCCTATACCCAC	GCTTGGCCACAGCACTATCA
*Gstm1*	NM_010358.5	GGGATACTGGAACGTCCGC	GCTCTGGGTGATCTTGTGTGA
*Sod2*	NM_013671.3	CAGGATGCCGCTCCGTTAT	TGAGGTTTACACGACCGCTG

## Data Availability

All the data are available via this manuscript.

## Supplementary Materials

The following supporting information can be downloaded at: https://www.mdpi.com/article/10.3390/antiox12122129/s1, Figure S1: The SD-OCT images provide detailed cross-sectional views of the retina, allowing visualization of retinal layers and structural changes from untreated and treated mice (4 different mice) from both eyes (Scale bar 75 micron); Figure S2: Effect of buspirone on Antioxidant mRNA Expression in normal ARPE-19 Cells without and oxidative damage. The figure depicts the relative fold change in mRNA expression levels of antioxidant genes in ARPE-19 cells following 24 h of treatment with buspirone. Cells were treated with three different concentrations: Control (untreated), 1 µM (micromolar), and 10 µM (micromolar). Quantitative real-time polymerase chain reaction (qPCR) was employed to assess the mRNA expression of key antioxidant genes in each treatment group. This result highlighted the dose-dependent effects and observed elevated trends in the mRNA expression levels. Notable antioxidants gene examined include; (A) GSTM1: Glutathione S-Transferase Mu 1, (B) CAT: Catalase, (C) HO1: Heme Oxygenase 1, (D) SOD2: Superoxide Dismutase 2, (E) MT1: Metallothionein 1, (F) NRF2: Nuclear Factor (Erythroid-Derived 2)-Like 2. The expression levels were normalized to Glyceraldehyde 3-Phosphate Dehydrogenas (GAPDH) for internal control. Statistical significance was determined using unpaired Student *t*-test (n = 4–6), with *p* < 0.05 considered as statistically significant. Significant differences indicated by asterisks.
