# Supplementary material for: Buspirone Enhances Cell Survival and Preserves Structural Integrity during Oxidative Injury to the Retinal Pigment Epithelium
Source: Antioxidants (Basel). 2023 Dec 17;12(12):2129. doi: 10.3390/antiox12122129 (PMC10740916; doi:10.3390/antiox12122129)
Supplement: Supplementary file 1 [file antioxidants-12-02129-s001.zip › antioxidants-2707999-supplementary.pdf]

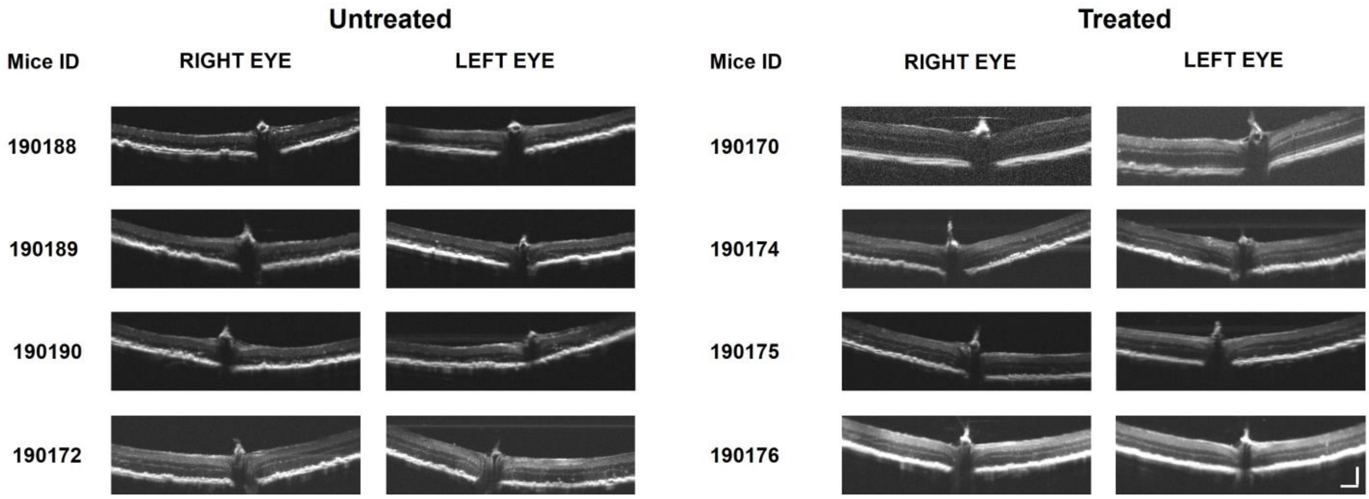

Figure S1: The SD-OCT images provide detailed cross-sectional views of the retina, allowing visualization of retinal layers and structural changes from untreated and treated mice (4 different mice) from both eyes.

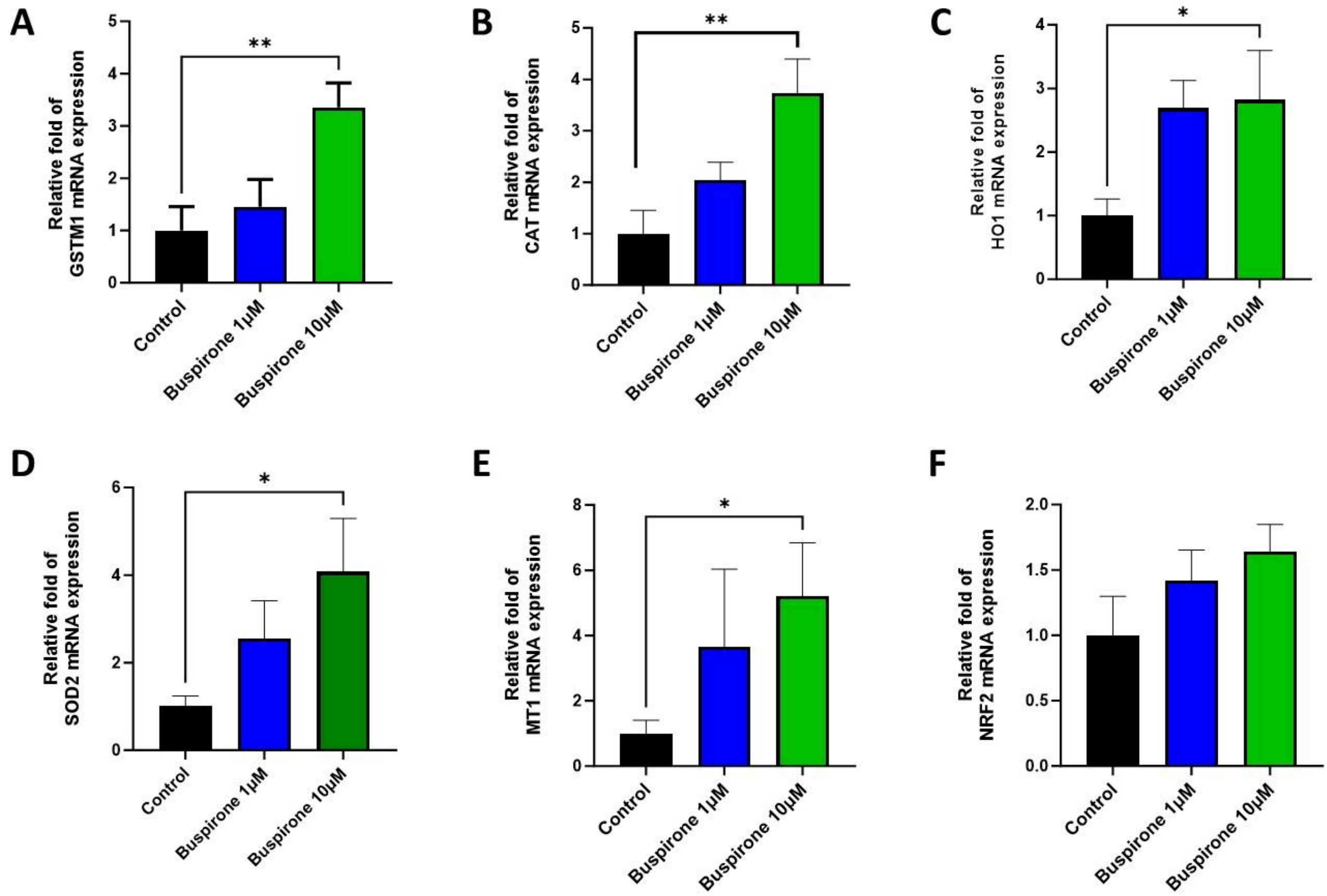

Figure S2: Effect of Buspirone on Antioxidant mRNA Expression in normal ARPE-19 Cells without and oxidative damage. The figure depicts the relative fold change in mRNA expression levels of antioxidant genes in ARPE-19 cells

following 24 hours of treatment with Buspirone. Cells were treated with three different concentrations: Control (untreated), 1 μM (micromolar), and 10 μM (micromolar). Quantitative real-time polymerase chain reaction (qPCR) was employed to assess the mRNA expression of key antioxidant genes in each treatment group. This result highlighted the dose-dependent effects and observed elevated trends in the mRNA expression levels. Notable antioxidants gene examined include; (A) GSTM1: Glutathione S-Transferase Mu 1, (B) CAT: Catalase, (C) HO1: Heme Oxygenase 1, (D) SOD2: Superoxide Dismutase 2, (E) MT1: Metallothionein 1, (F) NRF2: Nuclear Factor (Erythroid-Derived 2)-Like 2. The expression levels were normalized to Glyceraldehyde 3-Phosphate Dehydrogenas (GAPDH) for internal control. Statistical significance was determined using unpaired student t-test (n=4-6), with $p<0.05$ considered as statistically significant. Significant differences indicated by asterisks.
